# Study on the Mechanism of Jiaotai Pill Intervention on Insomnia Animal Model Based on Gut Microbiome and Metabolomics

**DOI:** 10.1155/2023/2442505

**Published:** 2023-05-23

**Authors:** Yang Yang, Jiao Liu, Haosong Ou, Xin Ma, Jia Li, Binghao Shao, Ruyi Jin, Junyun Zhao

**Affiliations:** School of Life Sciences, Beijing University of Chinese Medicine, No. 11 East Road, North 3rd Ring Road, Beijing 100029, China

## Abstract

**Background:**

With the continuous advancement of clinical application and experimental research of JTP, the application prospect of JTP in nervous system diseases and metabolic diseases is becoming increasingly clear. Jiaotai Pill (JTP) is a traditional Chinese medicine formula for insomnia, consisting of *Coptidis rhizoma* and *Cinnamomi cortex*, which dates back to *Han Shi Yi Tong* in the Ming Dynasty of China.

**Objective:**

Based on the brain-gut axis theory, this paper aims to explore the potential mechanism of JTP in the intervention of insomnia by using intestinal microbiome and metabolomics technology, taking the animal model of insomnia as the research object, so as to provide experimental basis for its further application and research.

**Methods:**

The insomnia mouse model was induced by intraperitoneal injection of para-chlorophenylalanine (PCPA). The clinical equivalent dose of JTP was administered by gavage for one week. The efficacy of JTP was evaluated by behavioral tests, serum biochemical detection, and brain histomorphological observation. The contents of cecum were analyzed by microbiomics and metabolomics.

**Results:**

The results show that insomnia caused by PCPA led to daytime dysfunction, higher HPA axis hormone levels, and morphologically impaired hippocampus. JTP reversed these anomalies. Omics research indicates that JTP significantly reduced gut *α* diversity; at the phylum level, JTP reduced the relative abundance of *Firmicutes, Deferribacterota, Cyanobacteria,* and *Actinobacteriota* and increased the relative abundance of *Verrucomicrobiota, Proteobacteria,* and *Desulfobacterota*. At the genus level, JTP reduced the relative abundance of *Muribaculaceae*, *Lachnospiraceae_NK4A136_group*, *Alistipes*, *Colidextribacter*, *Muribaculum*, and *Mucispirillum* and increased the relative abundance of *Bacteroides* and *Akkermansia*. JTP also reversed the activation of the linoleic acid metabolism pathway induced by insomnia. The combined analysis of omics suggests that JTP may play a role by regulating the inflammatory state of the body. Further gene expression analysis of brain tissue confirmed this.

**Conclusions:**

We hypothesize that JTP may achieve insomnia relief by eliminating inflammation-causing bacteria in the gut and reducing inflammation levels through the brain-gut axis, pointing to potential targets and pathways for future research on JTP.

## 1. Introduction

Insomnia is a condition that makes it difficult to fall asleep despite having the conditions for sleep and can interfere with daytime work with a prevalence of 19∼50% in adults [1, 2]. Insomnia increases the chances of depression [3], Alzheimer's disease, diabetes, Cardiovascular disease, hypertension, and dementia [1, 4]. Currently, cognitive behavioral therapy (sleep restriction, stimulus control, cognitive therapy, relaxation therapy, and sleep hygiene) is the preferred treatment for insomnia, and if it is not effective, then pharmacotherapy (benzodiazepine-receptor agonists, antidepressants, orexin antagonists, melatonin agonists, and anticonvulsant: gabapentin) is the preferred treatment for insomnia. However, all drug therapies have some side effects such as daytime sedation, dizziness, weight gain, and addiction [5].

Traditional Chinese medicine (TCM) has been used for more than 2000 years to treat insomnia and has the advantages of stability, nonaddiction, and nondependence [6]. Jiaotai Pill (JTP), a traditional TCM formula for insomnia, consists of *Coptidis rhizoma* and *Cinnamomi cortex* (Table 1), and its history can be traced back as far as *Han Shi Yi Tong* of the Ming Dynasty (韩氏医通) [7] and is currently used in the treatment of insomnia, depression, and type-2 diabetes [7, 8].

The brain-gut axis is one of the focuses in the study of nervous system diseases. The brain and gut communicate through three main channels (nervous system, endocrine system, and immune system). The existing experimental study of JTP shows that JTP could modulate the permeability of the gut barrier and microbiota in animal model of insomnia [7]. In brain tissue, JTP increases *γ*-aminobutyric acid levels and modulates the hypothalamic monoaminergic system and organic cation transporters [9]. In the intestine, JTP protects gut mucosa, regulates rhythm proteins [10], and decreases levels of lipopolysaccharides and inflammatory factors [7]. The effects of JTP on the brain-gut axis deserve further investigation.

## 2. Materials and Methods

### 2.1. Reagents

*Coptidis rhizoma* and *Cinnamomi cortex* are brought from Beijing Tong Ren Tang (Beijing, China). PCPA is brought from ARK Pharm (AK-80383, purity >98%). Mouse CRF ELISA kit (QS496683), Mouse ACTH ELISA Kit (QS43290), Mouse Cortisol ELISA Kit (QS43301), and Magen HiPure Total RNA Mini Kit (R4111-03) are from Guangzhou Meiji Bio-Technology (Guangzhou, China). RevertAid First Strand cDNA Synthesis Kit (K1621) is from Shanghai Jinpan Biotech (Shanghai, China). 2× PowerSYBR® Green PCR Master Mix (4367659) and PageRuler (26616) are from Thermo Fisher Scientific (MA, United States). RT-qPCR primers were designed by Primer Premier 5 software and commissioned to Sangon Biotech (Shanghai, China) for synthesis (Table 2). Tween 80 (T8360), RIPA (R0010), BCA protein concentration assay kit (PC0020), PMSF (P0100), ECL Plus hypersensitive luminescent solution (PE0010), hematoxylin eosin staining kit (G1120), sheep antirabbit IgG-HRP (SE134), horseradish peroxidase-labeled sheep antimouse IgG (SE131), and protein phosphatase inhibitor mixture (P1260) are from Beijing Solaibao Technology (Beijing, China); p38 MAPK polyclonal antibody (14064-1-AP), FOXO1 polyclonal antibody (18592-1-AP), TGF beta polyclonal antibody (19999-1-AP), and GAPDH polyclonal antibody (10494-1-AP) are from Proteintech (Chicago, USA). HPLC grade methanol and acetonitrile are from Merck (Dannstadt, Germany); formic acid and L-2-chloro-phenylalanine are from Sigma-Aldrich (MO, USA).

### 2.2. Drug Preparation and Composition Analysis

JTP was prepared as follows: referring to the method in the literature [11], *Coptidis rhizoma* and *Cinnamomi cortex* (10 : 1, w/w) were soaked in 10 times distilled water (w/v) for 1 h, refluxed twice for 1 h each, and the filtrates were combined and concentrated by spinning at 65°C. The filtrates were stored in portions at −20°C after it had been filtered aseptically by disc filters. They were thawed and mixed thoroughly at 4°C before use.

The composition of JTP was analyzed as follows: 30 ml of the prepared JTP solution was measured and lyophilized using an FD-2 freeze dryer (Beijing Biocool, Beijing, China), and the JTP-lyophilized powder was precisely weighed and prepared into a 2 mg/mL solution with ultrapure water, and then according to the method mentioned in the literature [12], the liquid mass spectrometry analysis was performed. The chromatographic conditions were as follows: chromatographic column: Eliot Supersil ODS2 column (2.1 mm *∗* 150 mm, 5 *μ*M); column temperature: 30°C; flow rate: 0.4 mL/min; injection volume: 2 *μ*L; mobile phase A: methanol; mobile phase B: 0.1% formic acid aqueous solution; detection wavelength: 254 nm; DAD detector (thermosScientific) gradient elution conditions: (0∼22.5 min, 21% A; 22.5∼35 min, 25% A; 35∼60 min, 60% A.). The mass spectrometry conditions were as follows: mass spectrometer: Q-Exactive Orbitrap quadrupole-electrostatic field orbitrap mass spectrometer. Ion source: thermal spray ion source (HESI), Xcalibur 4.1 chemistry workstation (thermo scientific). Positive ion scan mode; spray voltage: +3.5 kV, −3.0 kV; S-Lens RF voltage 55 V. Auxiliary gas flow rate: 10 arb; sheath gas flow rate: 35 arb; collision gas: helium; capillary temperature: 400°C; collision energy: 30, 35, 40; full scan (full scan, m/z 100∼1500), resolution: 70,000; data-dependent secondary mass spectrometry scan (data-dependent acquisition) ddMS2, resolution: 17500.

### 2.3. Animals and Treatments

Thirty ICR (Institute of Cancer Research) male mice, weighing 20 ± 2 g, were purchased from Beijing HFK Bioscience. The animals were housed in the animal room of Liangxiang Campus of Beijing University of Traditional Chinese Medicine at a room temperature of 22 ± 2°C, the humidity of 50 ± 5%, and a 12-hour day-night cycle, fed with mouse breeding chow (HFK Bioscience), and started the experiments after 1 week.

Mice were divided into three groups randomly: control group, PCPA group (450 mg/kg PCPA), and JTP group (450 mg/kg PCPA + 2.82 g/kg JTP) [8, 13], with 10 mice in each group. The mice were injected intraperitoneally at a dose of 450 mg/kg: 22.5 mg/mL of PCPA emulsion was injected into the PCPA and JTP groups once a day for 2 days; the mice in the CON group were injected with saline containing 5% Tween 80. PCPA emulsion was prepared as follows: PCPA was weighed precisely, added twice the weight of Tween 80, and ground thoroughly until there were no particles, then slowly saline was added and grinding was continued until completely emulsified. After PCPA was molded, the JTP group was gavaged with the aqueous decoction of JTP according to 0.2 mL/20 g, and the Con and PCPA groups were gavaged with distilled water according to the same volume twice a day for 7 days.

### 2.4. Behavioral Tests

The open-field test was conducted in the evening. The mice were first allowed to acclimatize in the experimental environment for 30 minutes, then the mice were gently placed in the center of the 60 *∗* 60 *∗* 50 cm open-field analysis box (UGO Baisle, Varese, Italy), and the total distance (horizontal distance + vertical distance) of mouse movement was tested for 5 minutes. At the end of the test, the chamber was wiped with 75% alcohol until the odor dissipated before starting the next mouse test, keeping quiet and light-free throughout the test.

The speed of the FT-200 animal treadmill (Chengdu Taimeng, China) was set to 12. Mice were gently placed into the runway, the cover of the treadmill was closed, and the number of times the mice were shocked within 3 minutes due to slow speed was recorded. At the end of the test, the runway was wiped with 75% alcohol until the odor dissipated and the next mouse test was started, keeping quiet throughout.

### 2.5. Sample Collection

Mice were fasted for 12 hours and anesthetized by intraperitoneal injection of sodium pentobarbital. Blood was collected by cardiac extraction and the serum was centrifuged at 3000 rpm for 10 min and stored at −20°C for backup. The contents of the mouse cecum were collected under aseptic conditions and immediately frozen and stored in liquid nitrogen, followed by transferring the cecum contents to an ultralow temperature refrigerator at −80°C for storage. The left brain of each mouse was fixed in fixative and the right brain was stored in −80°C ultralow temperature refrigerator.

### 2.6. ELISA

The serum of each group was collected and the levels of CRF, ACTH, and Cortisol were measured according to the kit instructions.

### 2.7. HE Staining

The tissue was dehydrated with gradient ethanol, paraffin-embedded and sectioned, dewaxed, and rinsed in deionized water three times for five minutes each for HE staining. The sections were again dehydrated with gradient ethanol and gradient xylene and sealed with neutral gum. They were observed under a Lecia Aperio Versa 8 super-resolution microscopic tissue imaging system (Oskar-Barnack-Straße, Germany).

### 2.8. Gut Microbiota Analysis

Eight mice from each group were randomly selected for gut microbiota 16S rRNA assay. The hypervariable region used for amplifying the 16S RNA gene is V3-V4. DNA was extracted from the contents of the mouse cecum using the DNeasy PowerSoil Kit (Unique, China) according to the kit instructions, and the purity of the extracted DNA was checked by agarose gel electrophoresis. After detection, PCR amplification was performed using Takara Ex Taq high fidelity enzyme and specific primers 343F: 5′- TACGGRAGGCAGCAG -3′ and 798R: 5′- AGGGTATCTAATCCT-3′ with barcode to ensure efficient and accurate amplification (94°C for 5 min; 94°C for 30 s, 56°C for 30 s, 72°C for 20 s, 26 cycles were performed; 72°C for 5 min; stored at 4°C). The purity of the PCR products was again checked by agarose gel electrophoresis, the magnetic beads were purified, PCR amplification was performed again and repeated twice, and the final products were quantified for concentration using Pultton P200/P200^+^ Nanodrop (USA), and the Illumina NovaSeq PE250 System (Santiago, USA) was used for sequencing.

### 2.9. Gut Metabolomics Analysis

The cecum contents of each of the 24 mice were mixed thoroughly, a certain amount of sample was weighed precisely, added with one-thousandth (v/w) of internal standard (0.3 mg/mL of L-2-chloro-phenylalanine, methanol configuration) and prechilled methanol and was ground thoroughly, and the mixture was extracted by ultrasonication in an ice-water bath for 30 min; chloroform was added, vortexed, ultrapure water was added, mixed well, and the new mixture was extracted again by ultrasonication in an ice-water bath for 30 min. The supernatant was extracted by centrifugation at 13000 rpm for 10 min at 4°C and loaded into glass derivatization vials; quality control samples (QC) were prepared by mixing equal volumes of the extracts of all samples. GC-MS metabolomics analysis was then performed.

The conditions for the liquid chromatography analysis were as follows: Agilent 6545 UHD and Accurate-Mass Q-TOF liquid chromatograph (CA, USA); Waters XSelect® HSS T3 column (100 mm × 2.1 mm, 2.5 *μ*m, Agilent), mobile phase A: 0.1% formic acid mobile phase A: 0.1% formic acid aqueous solution; mobile phase B: 0.1% formic acid acetonitrile; flow rate: 0.4 mL/min; injection volume: 4 *μ*L; gradient elution conditions: 0–3 min, 20% B; 3–9 min, 20–95% B; 9–13 min, 95% B; 13–13.1 min, 95-5% B; 13.1–16 min, 5% B. Positive and negative cosweep; capillary voltage. (+) 4.5 kV, (−) 3.5 kV; spray voltage: 120 V; Level 1 full scan (full scan, m/z 50∼1500), and the rest settings are instrument default.

### 2.10. RT-qPCR

Total RNA was extracted from the mouse cerebral cortex using Magen HiPure Total RNA Mini kit. Reverse transcription was performed using RevertAid First Strand cDNA Synthesis Kit. Each reverse transcription reaction (20 *μ*L in total) consisted of 1 *μ*g of RNA. Reactions were performed in a GeneAmp® PCR system 9700 (Thermo Fisher Scientific) at 42°C for 60 min; 25°C for 5 min, followed by stopping at 4°C. The relative expression of Il-1a, Il-6, and Il-*β* was detected by real-time fluorescence quantitative PCR (qPCR). Using ABI Quantstudio™ qPCR (Thermo Fisher Scientific), a 10 *μ*L PCR reaction mixture consisting of 1 *μ*L cDNA, 5 *μ*L 2× PowerSYBR® Green PCR Master Mix (Thermo Fisher Scientific), 0.2 *μ*M each of forward, and reverse primers, was fixed to 10 *μ*L with DEPC water. The reaction conditions were 95°C for 10 min; 95°C for 15 s, 60°C for 1 min, 40 cycles. Three replicate wells were set up for each sample. At the end of the PCR cycle, we run the default melting curve analysis of the instrument.

### 2.11. Western Blot

Mouse cerebral cortex was placed into test tubes, protein extraction reagents (RIPA, PMSF, and protein phosphatase inhibitor mixture) were added, mouse cerebral cortex was lysed with the assistance of an IKA T10-basic minidisperser (Staufen, Germany), total protein was extracted, protein was quantified and then subjected to SDS-PAGE electrophoresis, it was transferred using PVDF membrane, and then it was a closure. After blocking, diluted antibodies against p38, FOXO1, TGF-*β*, and GAPDH were added and incubated overnight at 4°C. After washing the membrane, the corresponding secondary antibody was added and incubated for 1 h. The membrane was developed using ECL ultrasensitive luminescent solution exposed to the BIO-RAD ChemiDoc™ MP imaging System (CA, USA).

### 2.12. Statistical Analysis

All experiments were performed with at least three biological replicates, and results were expressed as (mean ± SEM). Significance of differences in behavioral indicators, serum ELISA, mRNA expression, and protein expression was analyzed using SPSS20. Data were analyzed for statistical differences by one-way ANOVA if they conformed to a normal distribution with homogeneous variance; if the data did not conform to a normal distribution, they were analyzed by using a nonparametric test. Images of behavioral indicators, gene expression, and protein expression were plotted by Graphpad Prism 8. *P*  <  0.05 was considered statistically significant.

## 3. Results

### 3.1. JTP Components

**Figure 1:**
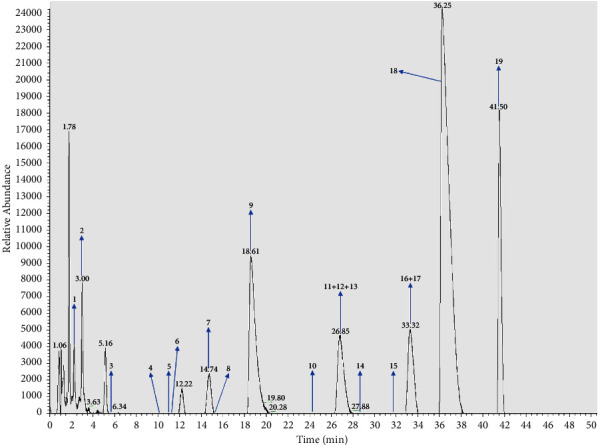
JTP ultra performance liquid chromatography (arrows mark the corresponding compounds).

The JTP components (Figure 1) were analyzed by UPLC-MS technique, and a total of 19 known components were found, including 8 from *Coptidis rhizoma* and 11 from *Cinnamomi cortex*, as detailed in the Table S1 (Supplementary Material).

### 3.2. Behavioral Analysis

**Figure 2:**
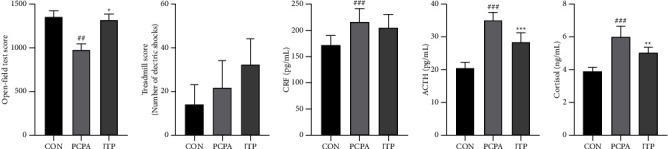
Behavioral and serum ELISA index assays. (a) Open-field test score: total distance of mouse movement (horizontal distance + vertical distance). (b) Treadmill test score: number of electric shocks due to slow running speed. (c–e) ELISA assays for HPA axis levels in mice serum. Compared with CON group: ^#^*P*  <  0.05, ^##^*P*  <  0.01; Compared with PCPA group, ^*∗*^*P*  <  0.05; ^*∗∗*^*P*  <  0.01, data expressed as mean ± SEM (*n* = 10).

Compared with the CON group, the PCPA group showed a significant decrease (*P*  <  0.01) in the total distance of activity in the evening; compared with the PCPA group, mice in the JTP group showed a significant increase (*P* <  0.05) in the total distance of activity in the evening. There was an increase in the number of shocks in the PCPA group compared to the CON group; compared to the PCPA group, the number of shocks showed an increase in the JTP group and the differences were not significant. The results are shown in Figure 2(a).

### 3.3. Levels of HPA Axis Hormones

As shown in Figures 2(b)–2(d), compared with the CON group, the PCPA group showed a significant increase in CRF, ACTH, and Cortisol levels (*P*  <  0.001); compared with the PCPA group, mice in the JTP group showed a regression in CRF levels and a significant decrease in ACTH and Cortisol levels (*P*  <  0.001 or *P*  <  0.01). The results suggest that JTP can reduce the levels of HPA axis-related hormones.

### 3.4. Brain Histomorphology

**Figure 3:**
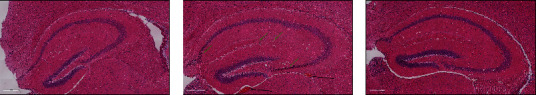
HE staining of the hippocampus in each group. (a) HE staining of hippocampus in the CON group; (b) HE staining of hippocampus in the PCPA group; (c) HE staining of hippocampus in the JTP group.

The HE staining of the hippocampus of each group is shown in Figure 3. The PCPA group had increased levels of inflammation and increased numbers of necrotic neurons compared to the CON group. The area indicated by the black arrow of the PCPA group in the figure shows highly dilated blood vessels, which are filled with a large number of erythrocytes, with inflammatory infiltration around the vessels, suggesting the development of inflammation. The neuronal cells in the PCPA group with green arrows show necrotic-like lesions, which are characterized by deepened eosinophilic staining and increased cytoplasmic eosinophilia; the nuclei are fixed and reduced in size or not visible. The number of necrotic cells in the PCPA group was higher than that in the CON group, and the surrounding space was larger than that in the CON group. The JTP group had decreased levels of inflammation and decreased numbers of necrotic neurons compared to the PCPA group. A small number of erythrocytes could be seen in the JTP group, but they were significantly less than in the PCPA group (*P*  >  0.05), and there was no inflammatory infiltration, suggesting that the level of inflammation in the JTP group was lower than that in the PCPA group. Neuronal necrosis occurred less frequently than in the PCPA group, and each necrotic neuron was more superficially eosinophilic and had a smaller necrotic area.

### 3.5. Gut Microbiota

A total of 3,372,976 reads were obtained from the gut microbiota of 24 mice after high-throughput sequencing. In order to study the species diversity of the samples, feature information was generated by DADA2. For the convenience of understanding and mapping, each feature obtained in this experiment was defined as an ASV (amplicon sequence variant). To obtain the species classification information, the reference genome Silva-132 99% features, pruned to v3-v4 region sequences and trained to obtain the classifier using Qiime2 based on the Silva-132-99 database, v3-v4 region amplicon primers, after which the trained naive Bayes classifier was used to feature to obtain the corresponding species classification information, thus obtaining the community composition of each sample.

**Figure 4:**
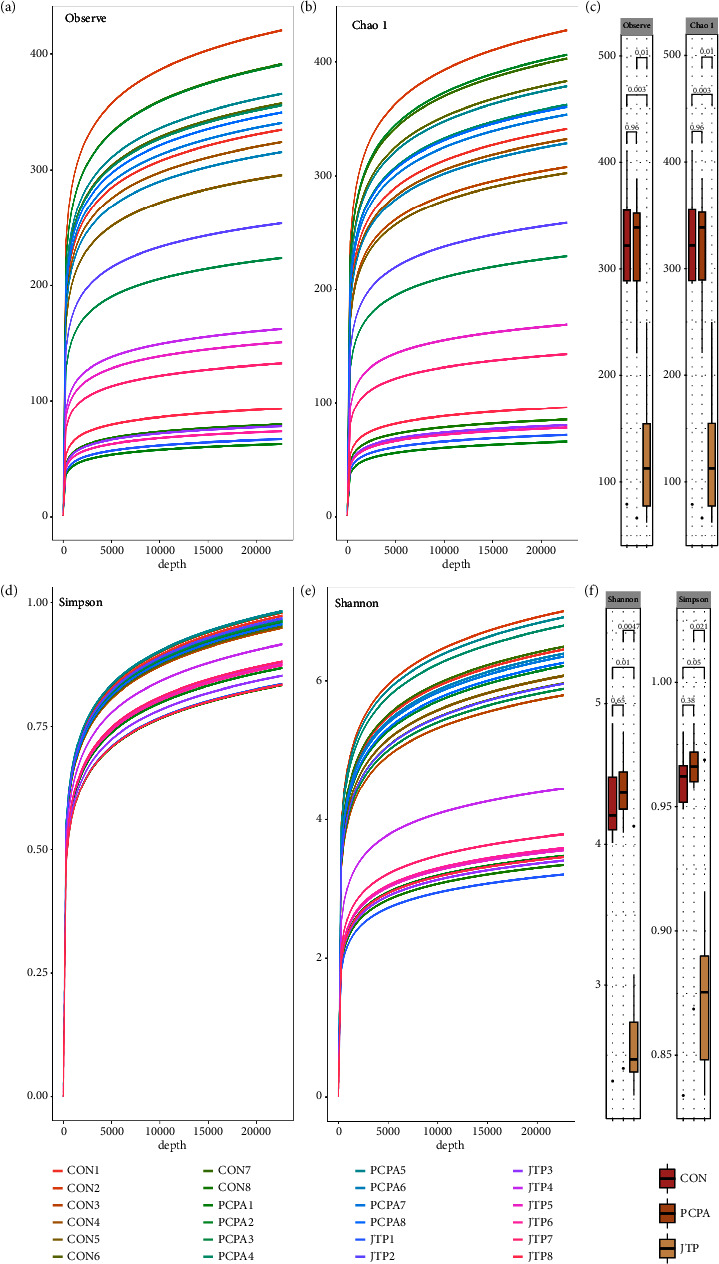
*α*-diversity analysis of the gut microbiota in each group (*n* = 8). (a) Observed analysis; (b) chao1 analysis; (d) simpson analysis; (e) shannon analysis; (c, f) significance analysis of each alpha diversity.

The alpha diversity was analyzed by Observe, Chao1, Simpson, and Shannon algorithms, and the curves have basically reached the plateau area, indicating that the sequencing depth has reached the basic requirements (as shown in Figures 4(a), 4(b), 4(d), 4(e)). In general, insomnia decreases the *α*-diversity of the gut microbiota [14]. However, such a result in the PCPA group was not observed but instead, a significant decrease in alpha diversity was observed in the JTP group, as shown in Figures 4(c) and 4(f). This result was verified by applying several algorithms to the analysis. The results showed that the gut microbiota alpha diversity was not significantly altered in the PCPA group compared to the CON group (*P*  >  0.05); the gut microbiota alpha diversity was significantly reduced in the JTP group compared to the PCPA group (*P* ≤ 0.01 or *P*  <  0.001). It indicates that JTP can reduce the gut microbiota *α*-diversity significantly.

**Figure 5:**
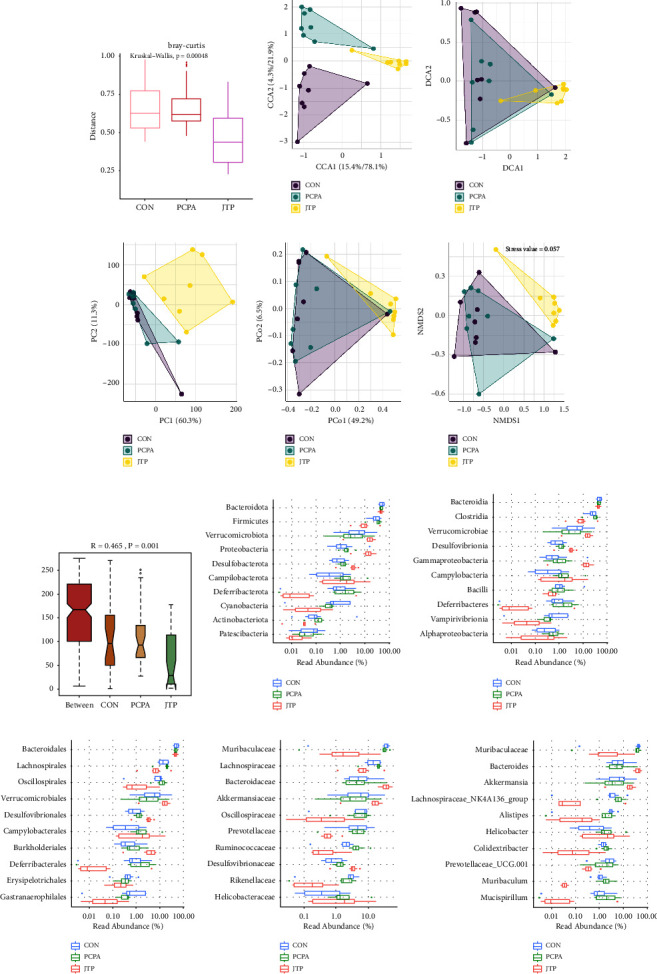
*β*-diversity analysis of the gut microbiota of each group (*n* = 8). (a) Bray–Curtis analysis; (b) CCA analysis; (c) DCA analysis; (d) PCA analysis; (e) PCoA analysis; (f) NMDS1 analysis; (g) ANOSIM analysis; (h–l) the top ten bacteria in each group of gut microbiota at five levels of phylum, class, order, family, and genus in terms of richness; each boxplot in the figure shows the minimum, first quartile, median, third quartile, and maximum values of each *α*-diversity index for samples within the group; the vertical coordinates represent the different species and the horizontal coordinates are the percentages of relative abundance.

Similar to the *α*-diversity of the mouse gut microbiota, the *β*-diversity of the microbiota in the JTP group also showed a significant trend of separation from the other two groups. As shown in Figure 5(a), the Kruskal–Wallis test results showed that the community differences between samples within each group were reproducible and not significant (*P* = 0.00048). The descending analysis is shown in Figures 5(b)–5(f). The NMDS1 result showed Stress = 0.057 < 0.2, indicating that the result can accurately reflect the degree of difference between samples. The CON and PCPA groups were not far apart, while the JTP group produced a significant separation from them. This indicates that the species composition of the CON and PCPA groups was similar, while the species composition of the JTP group was significantly different from that of the CON and PCPA groups. The CCA, DCA, PCA, and PCoA analyses all validated the NMDS1 results. As shown in Figure 5(g), the results of Anosim analysis showed that the between-group differences were greater than the within-group differences (*R* = 0.465 > 0), and the differences between groups were significant (*P*=0.001 < 0.05).

To further understand which bacteria's relative abundance was altered by JTP, the strains were ranked in order of relative abundance from largest to smallest, visually responding to differences in abundance between groups for the major strains, as shown in Figures 5(h)–5(l). At the phylum level, JTP reduced the relative abundance of *Firmicutes, Deferribacterota, Cyanobacteria,* and *Actinobacteriota* and increased the *Verrucomicrobiota, Proteobacteria,* and *Desulfobacterota*. The relative abundance of *Verrucomicrobiota, Proteobacteria,* and *Desulfobacterota* was increased. At the genus level, JTP reduced the relative abundance of *Muribaculaceae*, *Lachnospiraceae_NK4A136_group*, *Alistipes*, *Colidextribacter*, *Muribaculum*, and *Mucispirillum* and increased the relative abundance of *Bacteroides* and *Akkermansia*.

### 3.6. Gut Metabolites

**Figure 6:**
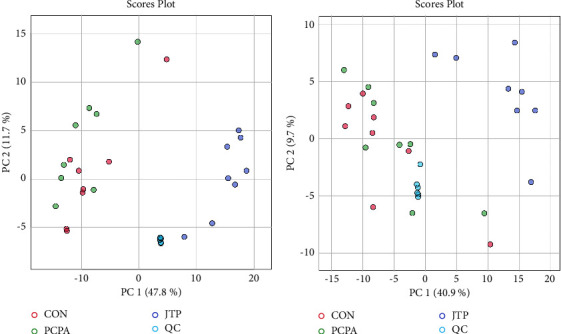
PCA score plots for each group of mouse cecum contents and QC samples (*n* = 8) (a) PCA score plots in positive ion flow; (b) PCA score plots in negative ion flow.

The PCA modeling method was used to examine the degree of aggregation of QC samples and then evaluate the quality of the experimental data. PCA analysis is an unsupervised modeling analysis method that can reliably reflect the most realistic differences between groups as well as reject outliers. As shown in Figure 6, it can be clearly seen from the figure that the QC samples are densely distributed, which indicates that the data quality of this experiment is reliable.

**Figure 7:**
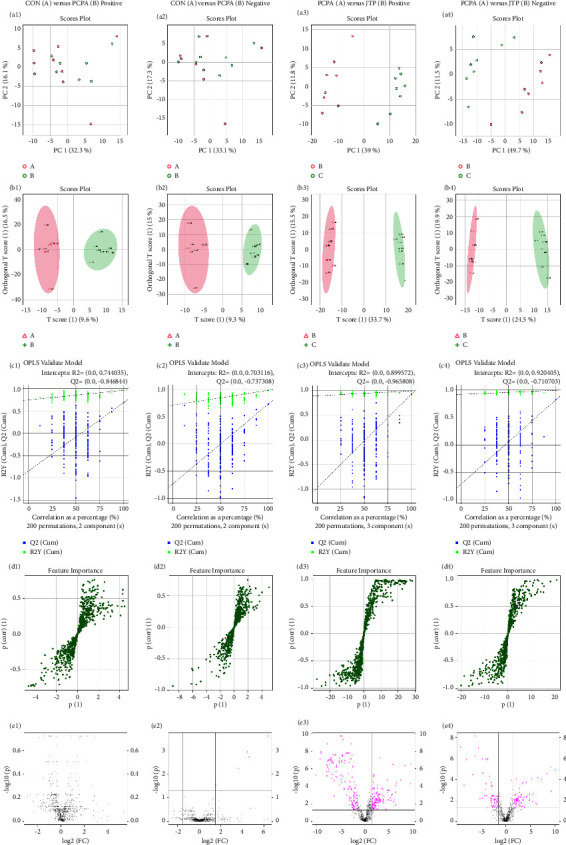
Metabolomic analysis of cecum contents (*n* = 8). (a1-a4) PCA analysis; (b1-b4) OPLS-DA analysis; (c1-c4) alignment test plots of OPLS-DA model; (d1-d4) S-Plot of OPLS-DA model; (e1-e4) volcano plots of differential metabolites.

In order to screen the differential substances between the groups, multivariate analysis method (PCA) and orthogonal projections were further used to latent structures discriminant analysis (OPLS-DA) for the different groups. The PCA and OPLS-DA score plots are shown in Figures 7 A1–B4, which show a clear separation trend between the PCPA and JTP groups but not between the CON and PCPA groups. In order to avoid “overfitting” the model, we performed the alignment test (Figures 7 C1–C4). It can be seen that the intersection of the line Q2 with the vertical coordinate is less than 0, which indicates that the model is not “overfitted.” The S-plot results of each group are shown in Figures 7 D1–D4. The points farther away from the origin indicate that their contribution to the difference between groups is greater, and their VIP (Variable Importance in the Projection) values are also greater. Volcano plots are shown in Figures 7 E1–E4. The criteria for determining the differential metabolites were FC > 1.5 and *P*  <  0.05, which can be visualized that the differential metabolites between the JTP and PCPA groups were significantly more than the differential metabolites between the CON and PCPA groups.

**Figure 8:**
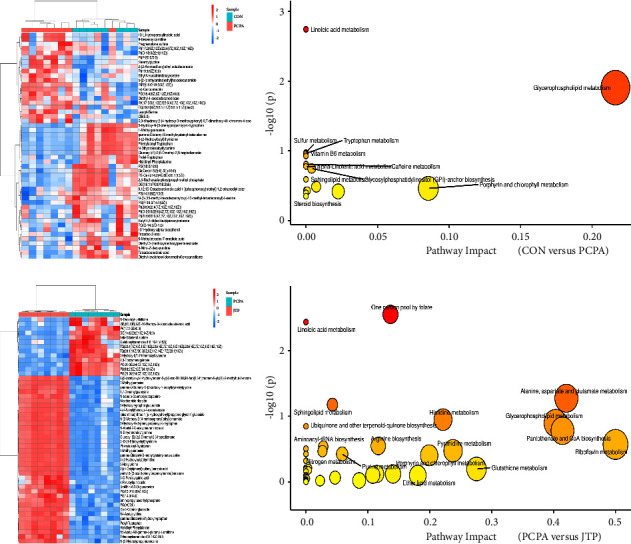
Differential metabolite heat map and differential metabolite pathway enrichment analysis scatter plot. (a) Heat map of differential metabolites between CON and PCPA groups; (b) bubble plot of differential metabolite pathway enrichment analysis between CON and PCPA groups; (c) heat map of differential metabolites between PCPA and JTP groups; (d) bubble plot of differential metabolite pathway enrichment analysis between PCPA and JTP groups.

The differential metabolites and their pathways were further analyzed. Differential metabolites were determined based on VIP ≥ 1 of the OPLS-DA model and independent samples *t* test (*P*  <  0.05). Metabolites were qualitatively identified by matching exact molecular weights to an online database, and masses under an error value of 30 PPM were considered as successful matches. Among them, [M+H]^+^ and [M+Na]^+^ were selected for the positive ion mode and [M−H]^−^ was selected for the negative ion mode when the online database was matched. The differential substances are shown in Figures 8(a) and 8(c). According to the differential metabolites, we used MetaboAnalyst online analysis software to analyze the differential metabolites in each group, and the pathway analysis species was selected as Mus musculus. The results of the pathway analysis are shown in Figures 8(b) and 8(d). The differential pathways in the CON and PCPA groups were linoleic acid metabolism and glycerophospholipid metabolism (*P*  <  0.05); PCPA and JTP groups differed in the following pathways: linoleic acid metabolism, one carbon pool by folate (*P*  <  0.05).

### 3.7. Transcriptional Levels of Inflammation-Related Genes

**Figure 9:**
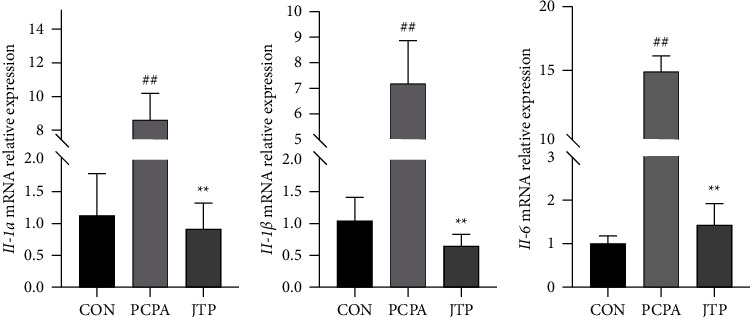
RT-qPCR to detect the transcript levels of Il-6, Il-1*α*, Il-1*β* in brain tissue. Compared with the CON group: ^#^*P*  <  0.05, ^##^*P*  <  0.01; Compared with PCPA group, ^*∗*^*P*  <  0.05; ^*∗∗*^*P*  <  0.01, data expressed as mean ± SEM (*n* = 3).

The mRNA level of Il-6, Il-1*α*, Il-1*β* in the PCPA group was significantly higher than the CON group (*P*  <  0.05 or *P*  <  0.01). Compared with the PCPA group, the mRNA levels of Il-6, Il-1*α*, Il-1*β* decreased in the JTP group (*P*  <  0.05 or *P*  <  0.01) (Figure 9).

### 3.8. Translation Levels of Inflammation-Related Genes

**Figure 10:**
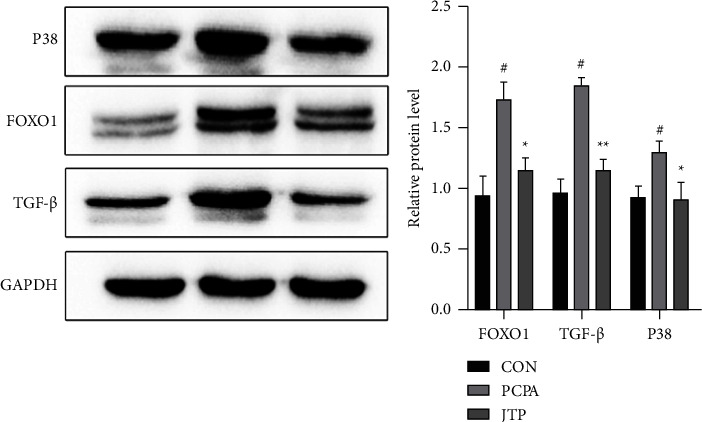
Western blot assay for protein expression levels of p38, FOXO1, TGF-*β* in brain tissue. Compared with the CON group: ^#^*P* <  0.05, ^##^*P* <  0.01; Compared with PCPA group, ^*∗*^*P*  <  0.05; ^*∗∗*^*P* <  0.01, data expressed as mean ± SEM (*n* = 3).

Compared with the CON group, the PCPA group showed a significant increase in the protein levels of FOXO1, TGF-*β*, and P38 (*P*  <  0.05). The protein level of FOXO1, TGF-*β*, and P38 in the JTP group was apparently lower than the PCPA group (*P*  <  0.05 or *P*  <  0.01) (Figure 10).

## 4. Discussion

As a traditional classic prescription, Jiaotai Pill is applied to the clinical treatment of insomnia for years. Experimental studies have confirmed the significant sedative-hypnotic effect of JTP [15]. However, its specific mechanism is still unclear.

In this paper, the open-field test and treadmill test were performed in the evening (the peak activity period of mice) to investigate the effect of JTP on autonomous activity and tension level. The result suggests that the improvement in open-field test scores in the JTP group was not through an increase in motor ability but possibly through an increase in willingness to explore by alleviating the anxiety-like symptoms caused by insomnia.

The levels of HPA axis hormones were significantly elevated in the PCPA group in this study, consistent with the clinical insomnia patients. Insomnia and the hypothalamic-pituitary-adrenal (HPA) axis interact with each other, activation of the HPA axis leads to lighter sleep and nocturnal awakenings [16], and insomnia, in turn, leads to activation of the HPA axis [17]. Higher HPA levels lead to increased cortisol levels throughout the day, and cortisol levels were positively correlated with the severity of insomnia [18]. The hormone level of JTP group decreased significantly, indicating that Jiaotai Pill can regulate the function of endocrine system and immune system.

In the brain tissue of the PCPA group, some cells had deepened cytoplasmic staining, suggesting neuronal cell necrosis; some regions were highly vasodilated, suggesting an increased level of inflammation in the brain tissue. This is consistent with hormonal changes in the HPA axis and confirmed by gene expression analysis of brain tissue later. Disruption of the HPA axis can impair hippocampal function and structure [19]. Excessive activation of the HPA axis leads to elevated glucocorticoid levels, which continuously activate glucocorticoid receptors in the hippocampus and damage hippocampal neurons [20]. Insomnia affects the inflammatory homeostasis of the nervous system, leading to the secretion of more proinflammatory factors by glial cells [21]. Glial cells responsible for the immune response of the nervous system are also densely distributed in the hippocampus [22]. A large number of cytokine receptors are present in the hippocampus, especially in the dentate gyrus [23]. IL-1*β* mRNA expression in the cerebral cortex and hippocampus increased significantly after 5 days of sleep restriction [24]. The decrease of neuronal necrosis and inflammatory cell infiltration in brain tissue in JTP group suggests that Jiaotai Pill can protect brain tissue function by regulating the inflammatory level of the body.

Gut microbiota analysis showed that the relative abundance of bacteria significantly altered in the JTP group. At the phylum level, the relative abundance of *Firmicutes, Deferribacterota, Cyanobacteria,* and *Actinobacteriota* decreased. The relative abundance of *Verrucomicrobiota, Proteobacteria,* and *Desulfobacterota* increased. A decline in F/B in JTP group was observed. The relative abundance of *Firmicutes* and *Bacteroidota* shows periodic oscillations throughout the day [25, 26]. Imbalance in the ratio of *Firmicutes* to Bacteroidota has been frequently observed in past insomnia studies. Sleep deprivation on two consecutive days would show a rise in F/B [27], and this acute insomnia is similar to the current modelling approach. However, some studies have also pointed out that insomnia is not associated with F/B or causes a decrease in F/B [14, 28, 29] and whether an increase in F/B is a feature of acute insomnia needs to be further investigated. F/B is widely associated with metabolism and that F/B increases with increasing body mass index BMI [30], leading to obesity and type-2 diabetes [31], which may be related to the fact that JTP is currently used mainly in the treatment of insomnia, depression, and type-2 diabetes. *Prevotellaceae* and *Muribaculaceae* are two major families of *Bacteroidota*, and *Ruminococcaceae* is an important family in *Firmicutes*. The altered relative abundance of these bacteria has significant circadian rhythms [32].

At the genus level, the relative abundance of *Muribaculaceae*, *Lachnospiraceae_NK4A136_group*, *Alistipes*, *Colidextribacter*, *Muribaculum*, and *Mucispirillum* decreased in the JTP group. The relative abundance of *Bacteroides* and *Akkermansia* increased. *Akkermansia* is a well-known probiotic, and numerous studies have shown the potential efficacy of *Akkermansia* in many diseases such as inflammatory diseases, metabolic syndrome, immune diseases, and cancer [33]. The relative abundance of *Akkermansia* in the JTP group was significantly higher than that in the PCPA group, suggesting that Jiaotai pill has potential therapeutic effect on inflammatory diseases and immune diseases. *Alistipes* has a pathogenic effect in anxiety, depression, inflammatory colon disease, and chronic fatigue syndrome and a protective effect on liver and cardiovascular fibrotic diseases [34]. *Lachnospiraceae* may contribute to inflammatory diseases such as metabolic syndrome, obesity, diabetes, liver disease, and depressive, and multiple sclerosis syndromes but also have beneficial effects such as short-chain fatty acid production and anti-inflammation in a healthy gut microbial environment [35]. The relative abundance of *Lachnospiraceae* in the JTP group was significantly lower than that in the PCPA group, suggesting that Jiaotai pill may reduce the possibility of inflammatory diseases. A few studies show that *Colidextribacter* is closely associated with liver fibrosis [36], serum oxidation levels elevation [37], and hyperlipidemia [38]. Whether observed at the phylum level or at the genus level, their altered relative abundance is more or less associated with various diseases, such as insomnia, depression, metabolic disease, and immune diseases.

The PCPA group did not show significant alterations in alpha diversity, instead, an extremely significant decrease in alpha diversity was observed in the JTP group, which may be related to the role of *Coptidis rhizoma* in JTP. In general, the alpha diversity of the gut microbiota decreases in insomnia patients [14]. *Coptidis rhizoma* showed activity of reducing alpha diversity, F/B, relative abundance of *Alistipes* and *Lachnospiraceae*, and increasing relative abundance of *Prevotellaceae UCG-001* [39, 40].

The effects of JTP on the mouse gut microbiota were mainly in the areas of remodeling bacteria with circadian rhythms, reducing inflammatory bacteria and enhancing probiotics. These changes in the relative abundance of microbiota may be one of the potential mechanisms for the efficacy of JTP.

Through metabolomics, the differential pathways were found. Linoleic acid (LA) can be converted into arachidonic acid in the body, which in turn can be converted into various active substances such as prostaglandins and leukotrienes, catalyzed by phospholipases. This leads to a series of chronic diseases, such as inflammation and cancer [41]. LA produces linoleic epoxides and leukotoxin diols, in the presence of CYP450 enzymes and soluble epoxide hydrolase. These LA metabolites modulate vascular permeability, stimulate neutrophil chemotaxis, and produce leukotoxicity [42]. Inflammation in the body also leads to increased LA metabolism [43]. The glycerophospholipid metabolic pathway is the main pathway for general systemic immunity and low inflammation, while phospholipids are also a potential mediator of inflammation [44]. One of the metabolites of the one carbon pool by folate pathway is folic acid [45], and excess folate makes white adipose tissue inflammation increased [46]. These results suggest that JTP may reduce inflammation by inhibiting pathways such as linoleic acid and one carbon pool by folate.

Significantly lower transcript levels of IL-6, IL-1*α*, and IL-1*β* and significantly lower protein translation levels of FOXO1, TGF-*β*, and P38 in the JTP group suggest that JTP may have a regulatory effect on inflammation in brain tissue. Sleep deprivation leads to an upregulation of mRNA expression of many immune-related genes (IL-1, TNF-*α*, IL-1*β*) [47, 48]. Immune responses can be involved in sleep regulation in the body, and in particular, proinflammatory cytokines such as IL-1 may promote the sleep through the hypothalamic nuclei [49]. IL-*α* and IL-1*β* are both cytokines that activate inflammatory processes, and their increased expression levels suggest inflammation [50]. IL-6 is rapidly and transiently expressed when the body perceives stress (infection and tissue damage), promoting the body to respond to stress through inflammatory and immune responses, and IL-6 production ceases when the stress disappears [51]. FOXO1 is a downstream gene of P38 that coregulate inflammation, for example, they can coactivate inflammatory vesicles leading to inflammation [52], and TGF-*β* is a class of multieffect cytokines that regulate cell differentiation, proliferation, and inflammatory response [53]. Molecular biological analysis of brain tissue indicates that JTP can improve the inflammatory state of brain tissue by regulating inflammation related pathways.

## 5. Conclusion

JTP may play its role in improving insomnia mainly by regulating the inflammatory level of the body (especially brain tissue), and the brain-gut axis plays an important role in this process. This study preliminarily explored the potential mechanism of JTP on insomnia animal model by using omics technology and focused on inflammation related pathways based on gut microbiota, gut metabolites, and analysis of gene expression in brain tissue. Because inflammation related pathways are closely related to the occurrence and development of many diseases, this paper not only explains the mechanism of JTP in the clinical treatment of insomnia from the perspective of regulating inflammation but also provides an important basis for the clinical application of JTP in other nervous system diseases, neurodegenerative diseases, and metabolic diseases. In the future, we will further explore the mechanism of JTP and its broad application prospects based on the in-depth mining of omics data.

## Figures and Tables

**Table 1:** Composition of the Jiaotai pill.

CHN pinyin	ENG name^a^	CHN name	Scientific name of plant^b^	Family name	Amount (g)
*Huanglian*	*Coptidis rhizoma*	黄连	*Coptis chinensis* Franch.; *Coptis deltoidea* C.Y. Cheng & P.G. Xiao; *Coptis teeta* Wall.	*Ranunculaceae* Juss.	15
*Rougui*	*Cinnamomi cortex*	肉桂	*Cinnamomum cassia* (L.) J. Presl	*Lauraceae* Juss.	1.5

^a^According to the Pharmacopeia of China 2015. ^b^According to the Tropicos v3.3.2 (Tropicos.org. Missouri Botanical Garden. 7 June 2022 <https://tropicos.org>).

**Table 2:** qPCR primer sequences.

Gene name	Forward primer (5′ ⟶ 3′)	Reverse primer (5′ ⟶ 3′)
Il-1*α*	GTTGCCAGAAACACCAAAAC	TGAATAGACTCCCGAAATAAGG
Il-1*β*	GAAATGCCACCTTTTGACAGTG	TGGATGCTCTCATCAGGACAG
Il-6	CCCACCAAGAACGATAGTCA	TCAGTCCCAAGAAGGCAAC
Gapdh	CCTCGTCCCGTAGACAAAA	GATGGCAACAATCTCCACTTT

## Data Availability

The original data supporting the findings of this study are available from the corresponding author upon request.

## Abbreviations

JTP: Jiaotai pill
PCPA: Para-chlorophenylalanine
RT-qPCR: Real time quantitative polymerase chain reaction
ELISA: Enzyme-linked immunosorbent assay
BCA: Bicinchoninic acid assay
HE: Hematoxylin-eosin
PCoA: Principal coordinate analysis
HPA: Hypothalamic-pituitary–adrenal
UPLC: Ultra performance liquid chromatography
P38: Mitogen-activated protein kinase 14
FOXO1: Forkhead box O1
TGF-*β*: Transforming growth factor, beta 1
GAPDH: Glyceraldehyde-3-phosphate dehydrogenase
CRF: Corticotropinreleasing factor
ACTH: Adreno-cortico-tropic-hormone
QC: Quality control
OPLS-DA: Orthogonal projections to latent structures discriminant analysis
RIPA: Radio-immunoprecipitation assay
PMSF: Phenylmethanesulfonylfluoride
ASV: Amplicon sequence variant
IL-6: Interleukin 6
IL-1a: Interleukin 1 alpha
LA: Linoleic acid
TNF-*α*: Tumor necrosis factor
IL-1*β*: Interleukin 1 beta
IL-1: Interleukin 1 complex.

## Supplementary Materials

Supplementary Materials Table S1: active compounds of JTP (Supplementary Material).
