# Supplementary material for: Study on the Mechanism of Jiaotai Pill Intervention on Insomnia Animal Model Based on Gut Microbiome and Metabolomics
Source: Evid Based Complement Alternat Med. 2023 May 23;2023:2442505. doi: 10.1155/2023/2442505 (PMC10229250; doi:10.1155/2023/2442505)
Supplement: Supplementary Materials — Table S1: active compounds of JTP (Supplementary Material). [file 2442505.f1.pdf]

TABLE S1: active compoun

No.	RT	$[M]^+$ Measured value	[M]+ Theoretical value	Error (ppm)	Proposed formula
1	2.25	382.19860	382.19931	-7.1	C20H30O7
2	3.00	342.16998	342.16965	3.3	C20H24NO4
3	5.65	562.26199	562.26215	-1.6	C26H42O13
4	10.14	408.21425	408.21390	3.5	C22H32O7
5	10.99	162.06753	162.06720	3.3	C10H10O2
6	11.34	352.11795	352.11728	6.7	C20H18NO5
7	14.74	322.10738	322.10709	2.9	C19H16NO4
8	15.36	352.22443	352.22406	3.7	C20H32O5
9	18.61	320.09173	320.09140	3.3	C19H14NO4
10	24.29	178.06245	178.06198	4.7	C10H10O3
11+12+13	26.38	132.05697	132.05702	-0.5	C9H8O
11+12+13	26.85	336.12303	336.12265	3.8	C20H18NO4
11+12+13	27.23	864.18962	864.19049	-8.7	C45H36O18
14	28.63	328.11527	328.11493	3.4	C15H20O8
15	31.77	134.07262	134.07286	-2.4	C9H10O
16+17	33.22	290.07849	290.07892	-4.3	C15H14O6
16+17	33.32	338.13868	338.13824	4.4	C20H20NO4
18	36.25	336.12303	336.12259	4.4	C20H18NO4
19	41.50	352.15433	352.15393	4.0	C21H22NO4

ds of JTP

Compounds
cinncassiol A
Magnoflorine
19-O-β-D-glucopyranoside
anhydrocinnzeylanine
cinnamaldehyde
13-hydroxyberberine
Dehydrocheilanthiflorine (or Berberrubine)
cinncassiol D4
Coptisine+Demethyleneberberine
coniferaldehyde
Cinnamaldehyde (or trans-cinnamaldehyde)
Epiberberine
B1,cinnamtannin D1
dihydromelilotoside
cinnamylalcohol
catechinicacid
Jatrorrhizine (or Columbamine)
Berberine
Palmatine
